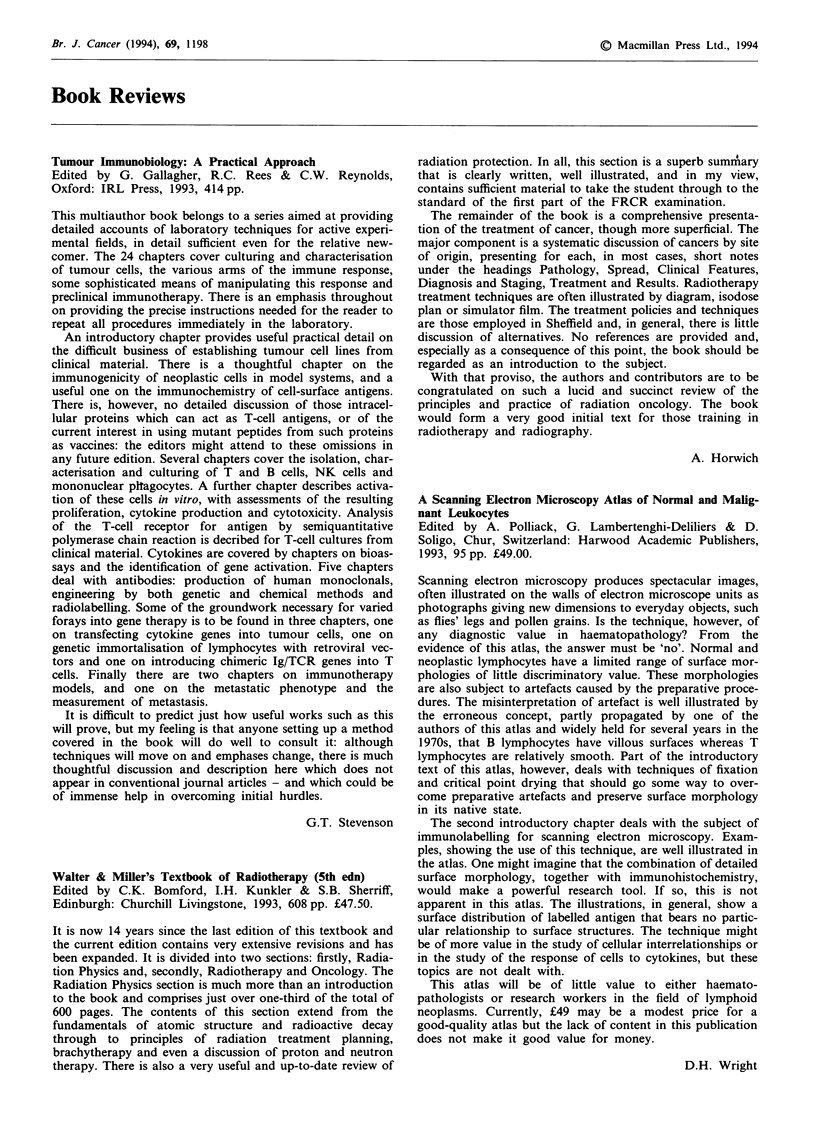# Tumour Immunobiology: A Practical Approach

**Published:** 1994-06

**Authors:** G.T. Stevenson


Br. J. Cancer (1994), 69, 1198 © Macmillan Press Ltd., 1994

### Book Reviews

**Tumour Immunobiology: A Practical Approach**
Edited by G. Gallagher, R.C. Rees & C.W. Reynolds, Oxford: IRL Press, 1993, 414 pp.

This multiauthor book belongs to a series aimed at providing detailed accounts of laboratory techniques for active experimental fields, in detail sufficient even for the relative newcomer. The 24 chapters cover culturing and characterisation of tumour cells, the various arms of the immune response, some sophisticated means of manipulating this response and preclinical immunotherapy. There is an emphasis throughout on providing the precise instructions needed for the reader to repeat all procedures immediately in the laboratory.

An introductory chapter provides useful practical detail on the difficult business of establishing tumour cell lines from clinical material. There is a thoughtful chapter on the immunogenicity of neoplastic cells in model systems, and a useful one on the immunochemistry of cell-surface antigens. There is, however, no detailed discussion of those intracellular proteins which can act as T-cell antigens, or of the current interest in using mutant peptides from such proteins as vaccines: the editors might attend to these omissions in any future edition. Several chapters cover the isolation, characterisation and culturing of T and B cells, NK cells and mononuclear phagocytes. A further chapter describes activation of these cells *in vitro*, with assessments of the resulting proliferation, cytokine production and cytotoxicity. Analysis of the T-cell receptor for antigen by semiquantitative polymerase chain reaction is decribed for T-cell cultures from clinical material. Cytokines are covered by chapters on bioassays and the identification of gene activation. Five chapters deal with antibodies: production of human monoclonals, engineering by both genetic and chemical methods and radiolabelling. Some of the groundwork necessary for varied forays into gene therapy is to be found in three chapters, one on transfecting cytokine genes into tumour cells, one on genetic immortalisation of lymphocytes with retroviral vectors and one on introducing chimeric Ig/TCR genes into T cells. Finally there are two chapters on immunotherapy models, and one on the metastatic phenotype and the measurement of metastasis.

It is difficult to predict just how useful works such as this will prove, but my feeling is that anyone setting up a method covered in the book will do well to consult it: although techniques will move on and emphases change, there is much thoughtful discussion and description here which does not appear in conventional journal articles – and which could be of immense help in overcoming initial hurdles.

G.T. Stevenson